# Depression Level and Its Associated Factors among Postpartum Working Women in Kuching, Sarawak—A Cross-Sectional Study

**DOI:** 10.21315/mjms2023.30.4.13

**Published:** 2023-08-24

**Authors:** Janting Majorie Ensayan, Whye Lian Cheah, Helmy Hazmi

**Affiliations:** Faculty of Medicine and Health Sciences, Universiti Malaysia Sarawak, Kota Samarahan, Sarawak, Malaysia

**Keywords:** postpartum depression, working mothers, physical symptoms

## Abstract

**Background:**

Postpartum depression (PD) among women, if left untreated, may result in long-term health and social consequences for them and their families. This cross-sectional study aimed to determine the factors contributing to PD among working mothers in Kuching, Sarawak, Malaysia.

**Methods:**

Systematic sampling was used to recruit working mothers who attended Kuching’s maternal and child health clinics. They were interviewed with a validated translated questionnaire to obtain data on sociodemographics, health profiles, and Edinburgh Postnatal Depression Scale (EPDS) and postpartum symptoms. Data were analysed using IBM SPSS version 21.0.

**Results:**

Out of the total 281 respondents, 15.3% of respondents had depression symptoms. Fatigue (42.7%), back or neck pain (36.3%), breast discomfort (16.4%), dizziness (13.5%) and nipple irritation (11.0%) were the most common physical symptoms experienced by the mothers. Regression analysis showed that working mothers who exhibited higher scores of physical symptoms were 1.26 times more likely to develop PD (adjusted odd ratio 1.26, *P* < 0.01; 95% CI: 1.071, 1.487).

**Conclusion:**

Physical symptoms were the predictors of PD among working mothers.

## Introduction

Women make up the majority of the labour force as they contribute up to 55.6% of the Labour Force Participation rate (1). Regardless of gender, employees are expected to perform in complex and competitive work environments to increase the productivity of their organisations (3). Mazlan et al. (4) reported that 16.9% of Malaysian workers reported that family life might affect the quality of their work life and female workers had a lower quality of work life compared to males. Many studies in the literature have highlighted the difficulties faced by working women in balancing their work life and family. Very often, parenthood increases the likelihood of women quitting their employment when family obligations become a priority for them (2). Women who get married and become pregnant often contemplate their future career pathways. Most working mothers are likely to return to work within a year after childbirth. It is crucial to support working mothers who resume work after childbirth to ensure that women are not left behind in labour force participation. Strong mental health and emotional fitness are vital to ensure good work performance and low absenteeism.

In addition, there is a significant concern about the mental health issues that affect women, especially in the postpartum period. Childbirth, generally, is supposed to be associated with great joy and happiness. However, the transition into parenthood can be overwhelming for new parents, occasionally leading to poor mental health among women during or after pregnancy (5). Depression experienced by mothers after childbirth, particularly during the first 4 weeks–6 weeks after birth can be debilitating. The postpartum period can go beyond 6 weeks and up to 6 months. The long-term consequences of postpartum depression (PD) can be devastating as they affect the functionality of the mother and also the wellbeing of her children, her partner, and other family members. It will also interfere with the child’s cognitive development and the maternal-child bond (6, 7). In addition, PD has been linked with absenteeism, poor work performance, and increased disability costs at the workplace.

PD is a depressive episode that occurs anytime during the first year after childbirth. It affects one in seven women (8). The World Health Organization (WHO) reported that about 20% of mothers in developing countries suffered from PD after childbirth. The prevalence of PD varies across countries between 4% and 38% despite using the same instrument (9). There are several local studies on PD among mothers (10, 11). However, none focused on working mothers with PD. It is possible that many factors influencing the development of depression between working mothers and non-working mothers may be different, thus this study aimed to determine the depression level and its associated factors among postpartum working women in Kuching, Sarawak, Malaysia.

## Methods

This was a cross-sectional study in Kuching, Sarawak, Malaysia. The study population consisted of working mothers who attended the Ministry of Health Malaysia’s Maternal and Child Health (MCH) clinics in Kuching. The sample size was calculated using PS software (12). Based on the prevalence of PD between 3.5% (13) and 14.3% (11) as well as a non-response rate of 30%, the total sample size required was 280. There are nine MCH clinics in Kuching, each with an average monthly attendance of 40 mothers. A total of 32 eligible mothers were recruited from each clinic. A systematic sampling method was used. The sampling frame was the patient attendance list provided by the clinics. Every other patient (even number on the list) was approached until the required sample size for each clinic was achieved. The inclusion criteria were full-time working mothers on maternity leave 6 weeks after delivery, and those seeking postnatal care at the clinics. Mothers with severe childbirth outcomes, severe obstetric conditions and pre-existing psychiatric illnesses were excluded from this study.

Data was collected using a three-part questionnaire. Part 1 focused on sociodemographic profile (age, ethnicity, parity and monthly family income), prenatal factors (chronic health problems, self-perceived prenatal health status and mood disturbances), postpartum factors (delivery complications, caesarean delivery, infant girl, breastfeeding and elapsed time after childbirth) and employment characteristics (employment status, occupational sector classification, job satisfaction and supervisory support). Prenatal health refers to the overall health status rated by the mothers as a whole before childbirth. For job satisfaction and prenatal mood disturbance, dichotomous responses were provided: whether they were satisfied or not with their job and whether there was prenatal mood disturbance (yes or no). Prenatal perceived control and supervisory support were measured using a Likert scale from none to complete and disagree to agree, respectively.

Part 2 was the Edinburgh Postnatal Depression Scale (EPDS), a 10-item questionnaire designed specifically to measure PD. It has been used extensively worldwide among mothers after childbirth (9). Each item is rated on a 4-point scale (0 to 3), with a total score ranging from 0 to 30. The Malay-translated version of this instrument was used (10, 14). Following the study by Azidah et al. (10), the cut-off point score of the Malay version of EDPS was 11.5, with 72% sensitivity and 92.6% specificity. Mothers with a score of 12 or higher were categorised as individuals with depression symptoms. The third part of the questionnaire was a 28-item postpartum symptom checklist adopted from McGovern et al. (15) and Gjerdingen et al. (16). It consists of a list of postpartum symptoms under six main categories, namely neurological symptoms, gynaecological and breast symptoms, cardiovascular and respiratory symptoms, skin and hair problems, gastrointestinal symptoms and general symptoms such as fatigue, fever, back and neck pain. Respondents were required to answer a ‘YES’ or ‘NO’ for each symptom in the checklist. A pre-test of the instrument was carried out on 30 respondents and the overall Cronbach’s alpha for EPDS was 0.850, indicating high reliability.

All potential mothers were approached after their postnatal check-ups or child immunisations at the MCH clinics. They were briefed about the purpose of the study and the questionnaire used. During the briefing process, voluntary participation and confidentiality were highlighted to the patients. They were also reminded that they were allowed to withdraw from the study at any time. After obtaining informed consent, they completed the questionnaire via a face-to-face interview with the researcher. Each session took about 20 min to complete. Ethical approval was obtained from the Medical Research and Ethics Committee of Universiti Malaysia Sarawak and the Ministry of Health Malaysia.

Data were entered and analysed using SPSS version 21.0. The univariate analysis consisted of Student’s *t*-test and Pearson’s chi-squared test were performed before multivariate logistic regression. A *P*-value lower than 0.05 was considered statistical significance in this study.

## Results

A total of 281 respondents participated in the study. The profile of the respondents is presented in Table 1. The majority of the respondents were Bumiputra Sarawak (42.7%). Most of the respondents had a minimum education level of secondary school. Almost half of the respondents were employed in the private sector (46.3%). About 96% of the respondents were satisfied with their job. Approximately 9.3% of working mothers claimed to have experienced prenatal mood disturbance before delivery. The EPDS score indicated that 15.3% of the respondents had depression symptoms.

In terms of the most common symptoms experienced by the mothers during the first 6 weeks after childbirth, Table 2 shows that fatigue (42.7%), back or neck pain (36.3%), breast discomfort (16.4%), dizziness (13.5%) and nipple irritation (11.0%) were the common symptoms.

Table 3 shows the association between respondents’ characteristics and PD. Mothers with scores lower than 12 on the EPDS were regarded as not having PD while scores of 12 and above EPDS indicated otherwise. The results showed that physical symptom score, prenatal mood disturbances, perceived job satisfaction and supervisory support were associated with PD during the first 6 weeks to 12 weeks after childbirth (*P* < 0.05). In this study, all mothers working in the government sector were highly satisfied with their job. While 7.5% of the working mothers (*n* = 21) scored higher than 12 on EPDS, mothers who worked in the private sector expressed dissatisfaction regarding their job. On the other hand, 6.8% of the working mothers (*n* = 19) scored higher than 12 on EPDS. Among the self-employed mothers, 1.1% of them (*n* = 3) expressed poor job satisfaction, thus indicating a higher risk of PD.

A multivariate logistic regression model was conducted to identify the predictors of PD (Table 4). Notably, no interaction occurred between the variables based on the multi-collinearity (MC) test before the multivariate logistic regression analysis. Furthermore, the variance-inflation factor (VIF) was lower than 10. Overall, the model’s goodness-of-fit was evaluated using the Hosmer-Lemeshow test. The *P*-value obtained was 0.236, thus indicating a good model fit. A precise prediction was made in 84.7% of the cases as to whether working mothers would experience PD or vice versa during the first 6 weeks after childbirth. Four factors in the initial analysis (Table 4) were identified as the significant predictors of PD, including perceived job satisfaction, supervisory support, prenatal mood disturbances and physical symptoms. However, in the final model, only physical symptoms experienced by the mothers remained a significant predictor (*P*-value < 0.005) of PD during the first 6 weeks after childbirth or postpartum.

Table 4 shows that working mothers who experienced more physical symptoms in the first 6 weeks after delivery faced a higher risk of developing PD. Those who experienced more significant physical complaints or exhibited higher physical symptom scoring were 1.3 times more likely to develop PD. The remaining factors such as prenatal mood disorder, perceived job satisfaction and supervisory support did not significantly affect the final multivariate analysis.

## Discussion

This study set out to determine the prevalence of PD and its association with physical symptoms among working mothers. The findings indicated fatigue as the most common physical symptom, followed by back and neck pain. These findings were consistent with studies conducted at different locations (17, 18) whereby the aforementioned symptoms were also identified as the most common symptoms. Fatigue or physical exhaustion experienced by postpartum mothers after childbirth can be attributed to several factors, specifically the process of childbirth and the transition into motherhood. Both of these factors are more prominent among first-time mothers. They often suffer from disrupted sleep as newborn babies can be light sleepers that wake up frequently throughout the night. Some mothers struggle with night-time feeding and settling the baby. Doering et al. (19) found that despite poor maternal sleep during the first few days after delivery, the condition improved with the baby’s nocturnal sleep pattern. Nevertheless, the prevalence of fatigue reported in this study was slightly lower than in two previous studies (15, 20, 21). The variation in the prevalence of fatigue among working mothers after childbirth could be influenced by other factors, including culture, surrounding support, spouse’s education level, and pre-existing sleep problems.

Based on EPDS, this study also found that 15.3% of the respondents were at risk of PD, a higher prevalence than Motzfeldt et al. (22) and Meijer et al. (23). One possible explanation was that the increased physical and emotional demands placed on new mothers in the postnatal period could have triggered depressive symptoms, subsequently impairing their capacity to take care of themselves, provide for the family and stay productive in their workplace. This is consistent with Frone et al. (24) who highlighted that working mothers are constantly in a dilemma because of the role conflict between work and family.

The univariate analysis showed that the work environment is an important aspect in the mothers’ life as they spend most of their time at the workplace every day. Furthermore, their job satisfaction relies heavily on various workplace factors such as the work environment, supervisory support, reward structure, and the flexibility for them to fulfill their family obligations (25). It was found that these factors were associated with PD in the early postpartum period. Furthermore, the two factors derived from the working environment, i.e. perceived job satisfaction and supervisory support were significantly associated with the development of PD during pregnancy until postpartum.

Overall, this study reported that workers in the government sector had lower job satisfaction as compared to self-employed individuals who had higher job satisfaction. Hence, there was also a higher number of mothers with PD among those working in the government sector when compared to other sectors. Self-employed individuals often report a higher level of job satisfaction (26). However, the findings in this study should be cautiously interpreted as the difference in job satisfaction can also be related to personality and time spent on job commitment. Notably, supervisory support and a good working environment can produce positive job satisfaction among the employees. The final regression model identified perceived job satisfaction and supervisory support as predictors of PD among working mothers during the first 6 weeks of the postpartum period.

Last but not least, self-perceived perinatal mood disturbances among working mothers were associated with the development of PD during the first 6 weeks of the postpartum period. Similarly, it was found in a previous study that any untreated disorders before pregnancy can cast a negative impact on mothers during and after pregnancy, ultimately affecting the wellbeing of the mother and the baby. Certain perinatal mood disorders such as anxiety disorders during pregnancy were associated with PD among mothers (27). However, the multivariate analysis failed to demonstrate any statistical significance in this matter. One possible explanation could be the different sub-populations as this study consisted of women of older age who were also working. In comparison, Allipour et al. (27) included younger individuals with a mean age of 22.8 years old (SD = 3.9 years old) who were predominantly housewives (97%). Therefore, it can be concluded that perinatal mood disorders were not predictors of PD among working mothers after childbirth.

Several limitations were found in this study. Ideally, the same screening tools should be used to evaluate the psychological wellbeing of mothers during antenatal and postnatal periods for a more objective comparison before they return to work after maternity leave. This is especially important because antenatal depression is another predisposing risk factor for PD (28). With this in mind, the exclusion of mothers with abnormal EPDS scores during pregnancy can produce a more convincing picture of PD among working mothers in future studies. The study findings can be used to improve healthcare services and other necessary interventions for working mothers after childbirth. In addition, the representativeness of the study samples in this study is another limitation. The generalisability of the study results was limited by the sampling method and sampling population as only working mothers in Kuching, Sarawak were recruited. Thus, the results might not be representative of other mothers in Malaysia. However, the results serve as a good baseline for future studies.

## Conclusion

This study assessed the PD experience of working mothers after childbirth. The results showed that physical symptoms were good predictors of PD among working mothers in the postpartum period. Generally, a higher number of symptoms experienced by the mothers led to a higher likelihood of developing PD. Overall, early assessment such as screening of physical health and timely intervention to alleviate the physical burden can reduce the risk of PD among working mothers.

## Figures and Tables

**Table 1:** Profile of respondent (*N* = 281)

	*N* (%)	Mean (SD)
Age (years old)		30.02 (4.82)
Ethnic		
Bumiputera Sarawak	120 (42.7)	
Malay	107 (38.1)	
Chinese	46 (16.4)	
Bumiputera Sabah	8 (2.8)	
Education level		
Primary to secondary	120 (42.7)	
Tertiary	161 (57.30)	
Household income (month, RM)		4,735.5 (3,957.78)
Occupation		
Government employees	123 (43.8)	
Private employees	130 (46.3)	
Self-employed/Own account worker	28 (10.0)	
Perceived supervisor’s support		
Agree	244 (86.8)	
Disagree	10 (3.6)	
Irrelevant	27 (9.6)	
Perceived job satisfaction		
Satisfied	270 (96.1)	
Unsatisfied	11 (3.9)	
Parity		1.9 (0.88)
Smoking	2 (0.7)	
Prenatal perceived control		
None/Very little	76 (27.1)	
A lot/Complete	205 (72.9)	
Chronic health problem	3 (1.1)	
Self-perceived prenatal health status		
Poor/Fair	1 (0.4)	
Good/Very good/Excellent	205 (99.6)	
Self-rated prenatal mood disturbance (depressed/anxious)	26 (9.3)	
Delivery complication	3 (1.1)	
Caesarean delivery	83 (29.5)	
Breastfeeding	273 (97.2)	
Depression level (EPDS scores)		6.26 (4.26)
< 12	238 (84.7)	
≥ 12	43 (15.3)	

**Table 2:** Physical symptoms occurring (*N* = 281)

Symptoms	*n* (%)	Mean (SD)
Physical symptom score		1.73 (0.16)
General		
Fatigue	120 (42.7)	
Back or neck pain	102 (36.3)	
Fever (> 37.8)	4 (4.14)	
Neurological		
Headache	15 (5.3)	
Hand numbness or tingling	17 (6.0)	
Gynaecological and breast		
Decreased desire for sex	22 (7.8)	
Breast discomfort	46 (16.4)	
Nipple irritation	31 (11.0)	
Breast irritation (mastitis)	–	
Uterine infection	2 (0.7)	
Cardiovascular		
High blood pressure	1 (0.4)	
Irregular heartbeat	–	
Respiratory		
Runny or stuffy nose	6 (2.1)	
Sore throat, cough, cold	7 (2.5)	
Sinus problems	1 (0.4)	
Skin and hair		
Acne	8 (2.8)	
Hair loss	24 (8.5)	
Excessive sweating	15 (5.3)	
Rash	6 (2.1)	
Gastrointestinal		
Constipation	19 (6.8)	
Abdominal pain	1 (0.4)	
Haemorrhoids	1 (0.4)	
Decreased appetite	1 (0.4)	
Diarrhoea	1 (0.4)	

**Table 3:** Association of respondents’ profile and scores of physical symptoms with PD at first 6 weeks to 12 weeks after childbirth

	EPDS scores				*P*-value

	< 12		≥ 12		
	
	Mean (SD)	*n* (%)	Mean (SD)	*n* (%)	
Age (years old)	29.9 (4.92)		30.7 (4.19)		0.332
Income (RM)	4,730.6 (4,172.65)		4,762.8 (2,485.58)		0.961
Physical symptom score	1.6 (1.89)		2.7 (2.08)		0.001^a^
Parity	1.9 (.90)		1.8 (.79)		0.661
Prenatal health^b^					
Poor/Fair		1 (0.4)		-	
Good/Very good/Excellent		237 (99.6)		43 (100.0)	
Ethnic					0.336
Bumiputera Sarawak		101 (42.4)		19 (44.2)	
Malay		92 (38.7)		15 (34.8)	
Bumiputera Sabah		5 (2.1)		3 (7.0)	
Chinese		40 (16.8)		6 (14.0)	
Education					0.803
Primary and secondary		101 (42.4)		19 (44.2)	
Tertiary		137 (57.6)		24 (55.8)	
Marital status^b^					
Single mother		13 (5.5)		1 (2.3)	
Married		225 (94.5)		42 (97.7)	
Prenatal perceived control					0.692
None/Very little/Some		67 (28.2)		9 (20.9)	
A lot/Complete		171 (71.9)		34 (79.1)	
Caesarean delivery					0.231
Yes		67 (28.2)		16 (37.2)	
No		171 (71.9)		27 (62.8)	
Breast feeding^b^					
No		8 (3.4)		-	
Yes		230 (96.6)		43 (100.0)	
Job satisfaction					0.005^a^
Satisfied		232 (97.5)		38 (88.4)	
Not satisfied		6 (2.5)		5 (11.6)	
Supervisor’s support					0.018^a^
Disagree		5 (2.1)		5 (11.6)	
Agree		123 (51.7)		22 (51.2)	
Strongly agree		86 (36.1)		13 (30.2)	
Irrelevant		24 (10.1)		3 (6.9)	
Occupational classification					0.670
Government		102 (42.9)		21 (48.8)	
Private		111 (46.6)		19 (44.2)	
Self-employed		25 (10.5)		3 (7.0)	
Prenatal mood disturbances					
Yes		18 (7.6)		8 (18.6)	0.039^a^
No		220 (92.4)		35 (81.4)	

Notes:

a significant at *P* < 0.05;

b insufficient cell count for analysis

**Table 4:** Logistic regression analysis for factors that predict PD in the first 6 weeks

	B	S.E	Wald	df	Sig	Exp (B)	95% CI for Exp (B)	

							Lower	Upper
Physical symptom score	0.233	0.084	7.744	1	0.005^a^	1.262	1.071	1.487
Prenatal mood	−0.510	0.519	0.966	1	0.326	0.601	0.217	1.660
Perceived job satisfaction	−1.406	0.803	3.066	1	0.080	0.245	0.051	1.183
Supervisor’s support	1.403	0.968	2.103	1	0.147	4.069	0.611	27.121

Notes: Model chi-squared = 37.13; *P* < 0.05; −2 log likelihood = 202.778; Pseudo *R*^2^ (Nagelkerke) = 0.236; the reference category is the PD group
